# Oyster hemolymph is a complex and dynamic ecosystem hosting bacteria, protists and viruses

**DOI:** 10.1186/s42523-020-00032-w

**Published:** 2020-04-28

**Authors:** S. Dupont, A. Lokmer, E. Corre, J.-C. Auguet, B. Petton, E. Toulza, C. Montagnani, G. Tanguy, D. Pecqueur, C. Salmeron, L. Guillou, C. Desnues, B. La Scola, J. Bou Khalil, J. de Lorgeril, G. Mitta, Y. Gueguen, J.-M. Escoubas

**Affiliations:** 1 IHPE, Univ. Montpellier, CNRS, Ifremer, Univ. Montpellier, Univ. Perpignan Via Domitia, 34095 Montpellier, France; 2 Coastal Ecology, Wadden Sea Station Sylt, Alfred Wegener Institute - Helmholtz Centre for Polar and Marine Research, List auf Sylt, Germany; 3 Current affiliation UMR 7206 Eco-anthropologie et Ethnologie, CNRS – MNHN Univ. Paris Diderot Sorbonne Paris Cité, Paris, France; 4 Sorbonne Université, CNRS, FR2424 ABiMS (Analysis and Bioanalysis for Marine Sciences), Station Biologique de Roscoff SBR, 29680 Roscoff, France; 5 MARBEC, Université Montpellier, CNRS, IFREMER, IRD, CC093, place Eugène Bataillon, 34095 Montpellier, France; 6 Ifremer, LEMAR UMR 6539, 11 presqu’île du Vivier, 29840 Argenton-en-Landunvez, France; 7 Sorbonne Université, CNRS, FR2424, Genomer, Station Biologique de Roscoff SBR, 29680 Roscoff, France; 8 Observatoire Océanologique de Banyuls sur Mer, FR 3724, BioPIC, CNRS/SU, Avenue Pierre Fabre, 66650 Banyuls-sur-Mer, France; 9 Sorbonne Université, CNRS, UMR7144 Adaptation et Diversité en Milieu Marin, Ecology of Marine Plankton (ECOMAP), Station Biologique de Roscoff SBR, 29680 Roscoff, France; 10 Aix-Marseille Université, IRD 257, Assistance-Publique des Hôpitaux de Marseille, UMR Microbes, Evolution, Phylogeny and Infections (MEPHI), IHU Méditerranée Infection, 13005 Marseille, France; 11 Aix-Marseille Université, Université de Toulon, CNRS, IRD, Mediterranean Institute of Oceanography, UM 110, 13288 Marseille, France; 12 Microbes, Evolution, Phylogeny and Infection (MEΦI), Aix-Marseille Université UM63, Institut de Recherche pour le Développement IRD 198, Assistance Publique – Hôpitaux de Marseille (AP-HM), Marseille, France; 13 Institut Hospitalo-Universitaire (IHU) - Méditerranée Infection, Marseille, France

**Keywords:** Oyster genetic background, Hemolymph microbiota dynamics, Early-life microbiota, Trans-kingdom interactions, *Crassostrea gigas*, Within-host ecosystem

## Abstract

**Background:**

The impact of the microbiota on host fitness has so far mainly been demonstrated for the bacterial microbiome. We know much less about host-associated protist and viral communities, largely due to technical issues. However, all microorganisms within a microbiome potentially interact with each other as well as with the host and the environment, therefore likely affecting the host health.

**Results:**

We set out to explore how environmental and host factors shape the composition and diversity of bacterial, protist and viral microbial communities in the Pacific oyster hemolymph, both in health and disease. To do so, five oyster families differing in susceptibility to the Pacific oyster mortality syndrome were reared in hatchery and transplanted into a natural environment either before or during a disease outbreak. Using metabarcoding and shotgun metagenomics, we demonstrate that hemolymph can be considered as an ecological niche hosting bacterial, protist and viral communities, each of them shaped by different factors and distinct from the corresponding communities in the surrounding seawater. Overall, we found that hemolymph microbiota is more strongly shaped by the environment than by host genetic background. Co-occurrence network analyses suggest a disruption of the microbial network after transplantation into natural environment during both non-infectious and infectious periods. Whereas we could not identify a common microbial community signature for healthy animals, OsHV-1 μVar virus dominated the hemolymph virome during the disease outbreak, without significant modifications of other microbiota components.

**Conclusion:**

Our study shows that oyster hemolymph is a complex ecosystem containing diverse bacteria, protists and viruses, whose composition and dynamics are primarily determined by the environment. However, all of these are also shaped by oyster genetic backgrounds, indicating they indeed interact with the oyster host and are therefore not only of transient character. Although it seems that the three microbiome components respond independently to environmental conditions, better characterization of hemolymph-associated viruses could change this picture.

## Background

Since the proposition of the hologenome theory of evolution [1], the majority of microbiome-related research has focused on its prokaryotic component (for review see [2]). However, recent findings indicate that we should also consider protists and viruses to understand the holobiont ecosystem (the host with its associated microbiota) and the role of microbiota in metaorganism functioning. For instance, the presence of protozoa correlates with higher diversity of the bacterial gut microbiome in humans, suggesting their potential beneficial role in the maintenance of homeostasis [3]. Similarly, the human body (including the circulatory system [4, 5]) is inhabited by highly diverse viral communities that can directly or indirectly influence the balance between health and disease [6], notably through their ability to modulate microbiota by infecting both bacteria and eukaryotes [7, 8]. On the other hand, the bacterial microbiota can shape the outcome of viral infections in mammals [9]. Nevertheless, the role and dynamics of host-associated non-bacterial microbiotas in invertebrates is understudied, with a few exceptions like corals [10, 11], hydra [12] or some insects [13–15].

One of the services that microbiota may provide to its host is the protection against pathogens. Numerous examples of microbiota-mediated protection span the tree of life and have recently inspired a concept of co-immunity, reflecting the increasing evidence that a host is protected not only by its own immune system, but also by its microbiota [16, 17]. On the other hand, disease is often associated with dysbiosis [18, 19] and a growing body of evidence supports a shift from a primarily “a pathogen - a disease” paradigm towards a more ecological view of disease (“a dysbiosis - a disease”), as reflected in the recently proposed “ecological Koch’s postulates” [20]. Overall, it is becoming clear that the understanding of a powerful selection pressure such as disease requires an understanding of the holobiont system.

In this respect, Pacific oysters (*Crassostrea gigas*) represent an interesting model for studying host-microbiome interactions, as for or more than a decade, oyster production has been plagued with a recurring mortality syndrome, called Pacific oyster mortality syndrome (POMS) [21]. POMS affects all coastal regions of France and numerous other producing countries [22, 23]. It coincides with the recurrent detection of *Ostreid herpesvirus* variants (OsHV-1 μVar) in moribund oysters both in France [24–26] and worldwide [22, 27–31]. In addition OsHV-1 μVar, POMS has been associated with various strains of the bacterial genus *Vibrio* [32]. Among these, populations of *Vibrio crassostreae* have been repeatedly identified in diseased oysters [33, 34]. We recently demonstrated that the disease is caused by polymicrobial infection, with the invasion of oyster hemocytes by OsHV-1 μVar as the initial and necessary step [35]. Altogether, these data highlight the need to simultaneously investigate the role of all hemolymph-associated microorganisms (bacteria, protists and viruses) in Pacific oyster health and disease.

So far, all studies regarding bivalve microbiota have focused on bacteria (reviewed in [36, 37]). The bacterial communities are tissue-specific: the microbiotas in oyster solid tissue differ from the hemolymph microbiota and both are distinct from the surrounding bacterial plankton communities [38]. The presence of living bacteria in the hemolymph of healthy bivalves [39–41] has been considered paradoxical, as the hemolymph contains hemocytes - circulating immune cells with phagocytic activity - and thus plays a key role in the oyster defense mechanisms [42]. However, it has been suggested that some of the resident hemolymph bacteria may contribute to oyster protection by producing antimicrobial peptides [43]. Although the number of descriptive studies of oyster bacterial microbiotas has been growing, few have addressed the factors shaping these communities [37]. Temperature seems to be a key driver of bacterial community structure and dynamics in oyster hemolymph [44], but many other host-intrinsic and environmental factors are potentially involved. Transplantation experiments conducted across different natural environments indeed suggested that the bacterial community structure and dynamics are influenced by a complex interplay of host-related factors, biotic interactions within the microbiota and local environmental conditions that require further exploration [45]. Finally, gill-associated bacterial communities are influenced by oyster genetics [46], but we know nothing about the influence of genetic factors on the hemolymph microbiota.

In this study, we aimed to explore how the environment, oyster genetic background and the hemolymph microbiota (bacteria, protists and viruses) interact with each other in the natural environment before and during a disease outbreak. Specifically, we aimed to: (i) describe the diversity and composition of oyster bacterial, protist and viral hemolymph microbiome, (ii) estimate the relative weight of host genetic background and environmental factors affecting the oyster blood microbiota composition in controlled as well as natural conditions (with low and high POMS risk), and (iii) detect co-occurrence patterns of microbes in oyster hemolymph and to examine their potential associations with the host health. By raising genetically distinct oyster families in controlled hatchery conditions, we were able to estimate the effect of host genetic backgrounds on the hemolymph microbiome, whereas transplantation experiments allowed us to examine the interactions between the environment, host genetic backgrounds and hemolymph microbiome in both health and disease.

## Methods

### Production of biparental oyster families

Five biparental oyster families were produced by in vitro fertilization from wild genitors sampled in farming and non-farming areas as previously described [35]. Briefly, wild genitors used to produce Atlantic families F9 and F15 were collected at two sites approximately 20 km apart, in Logonna Daoulas (lat.: 48.335263 long.: − 4.317922, farming areas) and Dellec (lat.: 48.353970 long.: − 4.566123, non-farming areas), respectively. Wild genitors used to produce Mediterranean families F32 and F44 were collected at two sites approximately 40 km apart, in Vidourle (lat.: 43.553906 long.: 4.095175, non-farming areas) and Thau lagoon (lat.: 43.418736 long.: 3.622620, farming areas) respectively. Genitors coming from aquaculture areas were assumed to be exposed to stronger selection pressure due to mass mortality outbreaks occurring annually at these sites. The last family, F21, was generated from a pair of broodstocks derived from mass selection conducted in the field during four generations in an aquaculture area at the Atlantic coast (La Tremblade, lat 45.781741 long − 1.12191, 450 km from the Mediterranean and around 370 km from the Atlantic sampling sites) [47]. In order to minimize environmental effects on hemolymph microbiota establishment, the oyster families were maintained in hatchery under controlled biosecured conditions before transplantation experiments.

### Transplantation experiments and sampling

In order to examine how oyster genetic background and environmental conditions affect the oyster microbiome in health and disease, we performed transplantation experiments before and during a mortality outbreak. Each of the five oyster families was divided into three batches (~ 300 oysters per batch); one was kept in the hatchery (at 16 °C) and the other two were transplanted for 5 days into an oyster farming area, the Thau lagoon (Lat. 43.378888 log. 3.571111), before and during a mortality outbreak. For transportation, oysters were packed in polystyrene containers and kept moist by covering with a damp cloth (duration of transport was less than 40 h). We previously showed that of 5 days of transplantation in the natural environment was sufficient to allow the oysters to be infected and precede massive mortality [48]. The non-infectious period (March 2016), was characterized by low average seawater temperature (10.6 °C), whereas the temperature during the infectious period (May 2016) was 18.7 °C. As microbiota may be affected by temperature stress [44], the oysters were progressively acclimated (2 °C/day) to Thau lagoon temperature before transplantation. Five days after the transplantation, the oysters were transported out of water to IHPE laboratory facilities located at 40 km, and hemolymph was drawn from the adductor muscle using a 1 mL syringe and 23 x ¼ needle. We sampled three replicates (of at least 30 oysters) per oyster family and per condition (hatchery, Thau non-infectious and Thau infectious). Hemolymph aliquots were kept on ice during the sampling. Hemocytes were removed from the hemolymph by filtration (5 μm membranes, Sartorius Minisart). Hemocyte-free hemolymph samples were filtered on 0.2 μm membranes, then the membranes and the filtrates were flash-frozen in liquid nitrogen and stored at − 80 °C. The membranes were subsequently used to extract DNA for bacterial and protist microbiota analysis. The filtrates were used to quantify virus-like particle and to extract DNA for viral microbiota analysis.

Seawater samples (10 L), were collected from each environment, filtered on 20 μm membranes to remove large particles in suspension and microorganisms collected by filtration as for hemolymph samples except that viruses contained in 0.2 μm filtrates were concentrated by *iron chloride flocculation* [49].

### DNA extraction and sequence processing for bacterial and protist metabarcoding

Total genomic DNA was extracted from 0.2 μm filters used to collect bacteria and protists from hemolymph and seawater using the NucleoSpin® Tissue Genomic extraction kit (Macherey-Nagel). Amplification and sequencing were performed by Genome Quebec Company (Genome Quebec Innovation Center, McGill University, Montreal, Canada). PCR amplifications were performed using primers 341F (5′-CCTACGGGNGGCWGCAG-3′) and 805R (5′-GACTACHVGGGTATCTAATCC-3′) targeting the V3-V4 region of the bacterial 16S rRNA genes [50]. For the V1-V2 region amplification of the eukaryotic 18S rRNA genes, we used the eukaryotic forward primer NEW EUK F (5′-ACCTGGTTGATCCTGCCA-3′) adapted from the EukA primer described by Medlin et al. [51] that we shortened by two nucleotides in 3′-end to catch more diversity. Based on a selection of complete sequences covering major eukaryotic lineages extracted from the PR2 database [52], we observed that this primer will target most eukaryotic lineages (93%), including oysters, with the notable exception of some metazoan (human for instance) and Microsporidia (Additional file 1: Table S1). The reverse primer NEW EUK R (5′-GTARKCCWMTAYMYTACC-3′) was newly designed with seven degenerated nucleotides for targeting major eukaryotic lineages excluding metazoans (especially oysters). Metazoans have ≥3 mismatches with this primer, with the notable exception of Cnidaria, Porifera and Ctenophora which have no mismatch (Additional file 1: Table S1). This primer has no mismatch with most of other eukaryotic lineages (about 77% of sequences based on our selection), with few exceptions, notably Parabasalia and Microsporidia. Paired-end sequencing with 250 bp read length was performed on a MiSeq system (Illumina) using the v2 chemistry according to the manufacturer’s protocol. Pre-process analyses and clustering was performed on FROGS pipeline (Find Rapidly OUT with Galaxy Solution) [53]. In brief, after denoising and primer/adapter removal with cutadapt, clustering was performed using SWARM, which uses a two-step clustering algorithm with a threshold corresponding to the maximum number of differences between two operational taxonomic units (OTU) (denoising step d = 1; aggregation distance = 3) [54]. To produce OTU and affiliation tables in the standard BIOM format, we performed an affiliation using Blast + against the Silva 16S rRNA database (release 128, Sept 2016) and the pr2 database (release 13, Sep 2017) for bacteria and protists respectively. OTU relative abundances and taxonomic affiliations of the bacteria and protists found in the seawater and the hemolymph are available in Additional file 2: Table S2 and Additional file 3: Table S3, respectively.

In order to verify that we did not introduce contamination during metabarcoding processes (from DNA extraction to sequencing), we made six independent DNA extractions blank controls using the NucleoSpin® Tissue Genomic extraction kit. As we hypothesized that the most probable source of contamination was of bacterial origin, amplification and sequencing were performed on these 6 samples using the primers targeting V3-V4 region of the bacterial 16S rRNA genes. At the end of the bio informatics analyses we obtained 15 OTUs and none of them was common to the six blank controls. As only two of these OTUs are found in some of the samples and represent on average 0.09% of the reads (45 reads/sample out of 50,000) and as we used abundance-weighted analysis methods, we considered that our DNA extraction and sequencing process do not introduce significant contamination.

### DNA extraction and sequence processing for viral metagenomics

Viral particles were collected from the 48 samples (45 hemolymph samples and 3 seawater) by ultracentrifugation (257,000 g for 2 h at 4 °C, Beckman Optima). The pellets were re-suspended in sterile 1× PBS and treated with DNase I (10 U/μL RQ1 DNAse, Promega). Viral DNA was extracted using NucleoSpin® Virus kit (Macherey Nagel), and DNA amplification was run with a GenomiPhi™ V3 Kit (GE Healthcare). DNA concentration was measured using Qubit dsDNA HS assay kit (Invitrogen). Genomes were fragmented (average size 1225 bp for the 48 samples, ranking from 814 bp to 2227 bp), amplified and sequenced on a MiSeq system at the Genomer platform at Station Biologique in Roscoff. The 48 viral libraries corresponding to 92,891,278 paired-end 300 bp sequences (2,109,133 paired-end reads per library +/− 904,066) were cleaned by adapter removal and trimmed of low quality bases using Trimmomatic [55]. To remove host sequences, cleaned reads were mapped (Bowtie 2) to the *C. gigas* genome (GCA_000297895.1). rRNA contamination was removed using SortMeRNA software [56]. Full metavirome was assembled using MetaSPAdes [57] and coverage virome analyses were performed using the Bowtiebatch and Read2RefMapper scripts developed within the iVirus protocol [58]. Raw demultiplexed sequence data are available at NCBI SRA under the accession number PRJNA381401.

Viral contigs were annotated using VirSorter [59], Phaster [60] and BLAST against the Global Ocean Virome database (E-value <1e^− 10^). We further employed a procedure that improves short scaffold annotation [61]. Briefly, open reading frames (ORFs) were predicted with Prodigal [62] and aligned against *nr* database using BLASTP (E-value <1e^− 5^). We considered an ORF to be of viral origin based on the best blast hit annotation (BBH), or - in the case of bacteriophages - if one of the top 10 hits was a phage protein with one of the following phage-related functions: tail, coat, head, capsid, portal protein. Viral contig abundance and taxonomic affiliation in seawater and hemolymph are available in Additional file 4: Table S4.

### VLP quantification and cell sorting

VLP (Virus-Like-Particle) abundances were determined for three 1.5 mL aliquots per sample by flow cytometry (adapted from [63]), using a FACSCanto II cytometer (Becton Dickinson, Franklin Lakes, NJ, U.S.A.) equipped with an air-cooled laser providing 15 mW at 488 nm with the standard filter set-up. Abundances of VLPs were calculated using specific calibrated Becton Dickinson Trucount™ beads. Samples were stained with SYBR Green I (S7563, Invitrogen; 2% final concentration) and incubated at 80 °C for 20 min. After cooling down to room temperature, 1 μL of mixed fluorescent 0.5 μm-diameter beads (Molecular Probes Inc., Eugene, OR, U.S.A.) were added as an internal standard to variable sample volume mixed with 0.02 μm filtered TE buffer [63] to reach a final dilution between 400 and 2000 times. The flow rate of the cytometer was set at low level (acquisition time: 1 min) and artificially induced fluorescence (FL1: 530 nm) was used to detect VLPs. The flow cytometric data were analyzed using the Diva software (Becton Dickinson).

### Rarefaction analysis and subsampling of bacterial and protist datasets

We performed a rarefaction analysis of Good’s coverage and alpha diversity indices (Shannon’s H, evenness, Chao 1 and Observed OTUs) in QIIME [64] to determine an adequate subsampling depth for the bacterial and protist datasets. We chose the cutoff of 50,000 reads per sample for bacteria and 8000 for the protists (two samples with 7000+ reads were included as they were). We found high Good’s coverage for the above cutoff values as well as leveling-off of the rarefaction curves for Shannon’s H and evenness (Additional file 5: Figure S1).

### Statistical analyses

All statistical analyses were performed in R [65] at the OTU level (or contigs for viruses). Alpha diversity was represented by Shannon’s H and Observed OTUs indices calculated in *mothur* [66] and analyzed by generalized least squares by maximum likelihood linear models implemented in the package *nlme* [67]. The variance structure was included in the model in case of the variance heterogeneity as determined by the Levene’s test from the package *lawstat* [68]. Beta diversity based on Bray-Curtis index was visualized by nonmetric multidimensional scaling - NMDS and statistically analyzed by Permanova, [69] using the package *vegan* [70].

For both alpha and beta diversity, we first compared the seawater and hemolymph microbiota in each environment. We subsequently focused on the hemolymph microbiota, fitting three different models. The first two models tested the differences between the families grouped either by genitors’ origin or selection pressure experienced by genitors in the hatchery and infectious environment, respectively. The third model tested for differences between the hatchery and each of the natural environments (i.e., hatchery vs. non-infectious and hatchery vs. infectious environment). In this third model, the families were included as random effects in case of alpha diversity or used as blocks to constrain permutations in case of beta diversity. For each model, we first checked if any of the tested factors were significant and, if this was the case, we subsequently inspected the differences between two sets of a priori defined orthogonal contrasts, genitors’ origin and selection pressure experienced by genitors (Additional file 6 Table S5). We also performed indicator species analysis (using R package *indicspecies,* [71]) on all three microbiotas in order to find taxa significantly changing with environmental conditions and depending on the family origin and phenotype (Additional file 7: Table S6). We included only taxa with mean relative abundance > 1% in the analysis, thus testing 2505 bacterial, 357 protist and 6788 viral taxa. We defined indicators as OTU/taxa with both specificity (component A of the index, probability that oyster belongs to the group if the indicator is found) and sensitivity (component B, probability that the oyster in the group hosts the indicator) ≥ 0.9.

Associations between bacterial and eukaryotic OTUs were inferred from an undirected co-occurrence network. Pairwise scores between OTUs were computed using Spearman’s rank correlations. Only co-occurrences corresponding to correlations with a coefficient (rho) > 0.7 and a statistical significance (*P*-value) < 0.001 were considered for further analysis. Non-random co-occurrence patterns were tested with the checkerboard score (C-score) under a null model preserving site frequencies [72, 73]. Network characterization for comparisons between hatchery, infectious and non-infectious environments (15 samples in each network) was performed using a set of overall network topological indices (i.e., average node connectivity, average path length, average clustering coefficient and modularity) [74, 75]. All analyses were run using the R packages *vegan* [70], *igraph* [76] and *WGCNA* [77].

## Results and discussion

### Transplantation experiments of oyster families with different resistance phenotypes to Pacific oyster mortality syndrome (POMS)

**Fig. 1:**
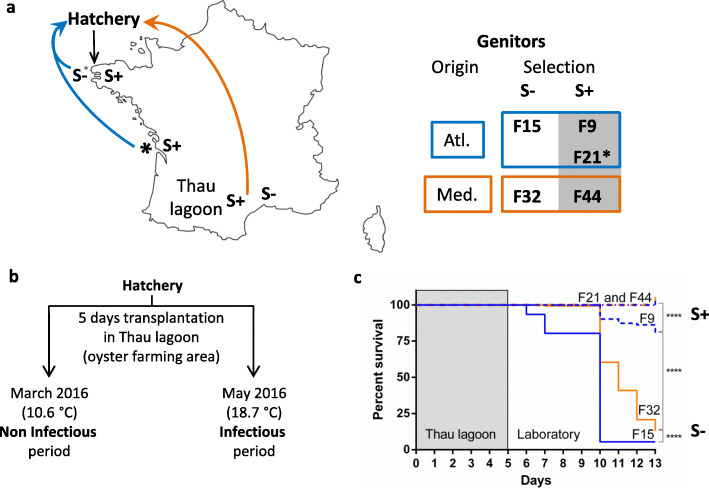
Production of oyster families with different level of resistance to Pacific oyster mortality syndrome (POMS). **a** Origin of genitors and production of the five biparental families. Four pairs of wild genitors were collected either in aquaculture (S+) or non-aquaculture (S-) areas on Atlantic (Atl.) or Mediterranean (Med.) coasts (blue and orange lines, respectively). The fifth pair of genitors (*) comes from a mass selection program (more details in method section). Genitors coming from aquaculture areas were assumed to be exposed to stronger selection pressure due to mass mortality outbreaks occurring annually at these sites. **b** Transplantation experiments. Oysters from the five families were raised in the hatchery under the same controlled conditions, and subsequently transplanted into the Thau lagoon either before (non-infectious period) or during a mortality outbreak (infectious period). **c** Survival curves for the five oyster families after transplantation into the Thau lagoon during the infectious period (a minimum of 125 oysters was used for survival tests). Day 5 corresponds to the end of the transplantation, when oysters were brought back to the laboratory. Resistant and susceptible oyster families are indicated by S+ and S-, respectively. **** *p* < 0.0001 (Mantel-Cox Log-rank test)

To investigate the role of the oyster genetic background and environment in the composition and dynamics of the hemolymph microbiota, we examined five genetically differentiated biparental oyster families with various POMS resistance phenotypes [35] (summarized in Fig. 1a) in controlled (hatchery) and natural (before and during disease outbreak) conditions (Fig. 1b). We observed mortality events only during the infectious period (Fig. 1c). The families produced from genitors collected in non-aquaculture areas had markedly lower survival rates (5.5 and 12.8% for F15 and F32, respectively) than the families from genitors collected in aquaculture areas (80.5% for F9 and 100% for F21 and F44). This difference justifies grouping the families into resistant (S+) and susceptible (S-), and supports our hypothesis that the selection pressure experienced by genitors determines the resistance to POMS.

### Hemolymph hosts a complex microbiota different from the environment and shaped by the oyster genetic background

**Fig. 2:**
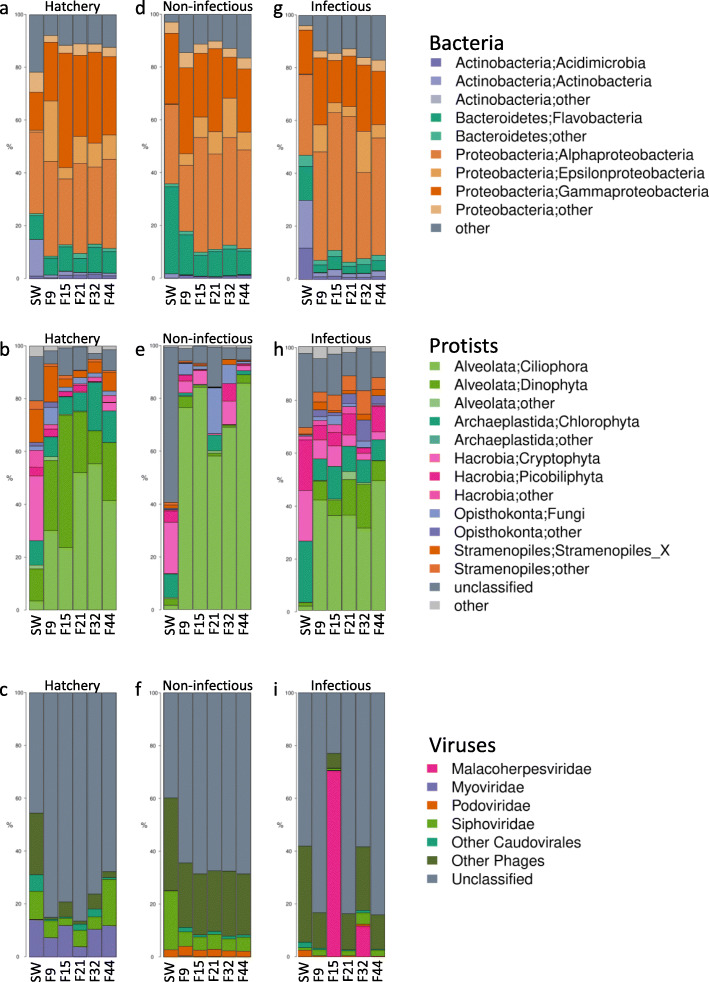
Relative abundance of bacterial classes, protistan phyla and viral taxa in oyster hemolymph and seawater in controlled and natural conditions. **a-c**) Hatchery, **d-f**) natural environment during non-infectious and **g-i**) infectious period. Bacterial classes and protistan phyla with relative abundance < 1% are grouped as “other”. The viral taxonomic affiliation was done on the 200 most abundant scaffolds, which represented 42.6% of the total reads (**c, f, i**). SW: seawater

Our results show that oyster hemolymph hosts a complex community of bacteria, protists and viruses, which differs from the microbial community in the seawater column not only at the OUT level (Additional file 8: Table S7), but also at higher taxonomic levels (Fig. 2a-c). Compared to the seawater from the hatchery tank, oyster hemolymph was characterized by a higher proportion of Proteobacteria (hemolymph: 78.1% ± 1.5 SEM, seawater: 53.4%, Fig. 2a) and Alveolata (hemolymph: 68.4% ± 3.5 SEM, seawater: 17.0%, Fig. 2b), and a lower proportion of Actinobacteria (hemolymph: 2.3% ± 0.2 SEM, seawater: 15%) and viruses of the “other phages” category (hemolymph: 3.1% ± 1.1 SEM, seawater: 23.3%, Fig. 2c). Differences in bacterial, protist and viral seawater and hemolymph communities were also observed after transplantation into a natural environment (Fig. 2d-i). The fact that the hemolymph and seawater microbiota differ already at a low taxonomic resolution indicates that the hemolymph is indeed a distinct environment. Whereas the distinctness of hemolymph bacterial microbiota has been previously reported [45, 78] here we also show that the hemolymph hosts rich and distinct protist and viral microbial communities as well.

**Fig. 3:**
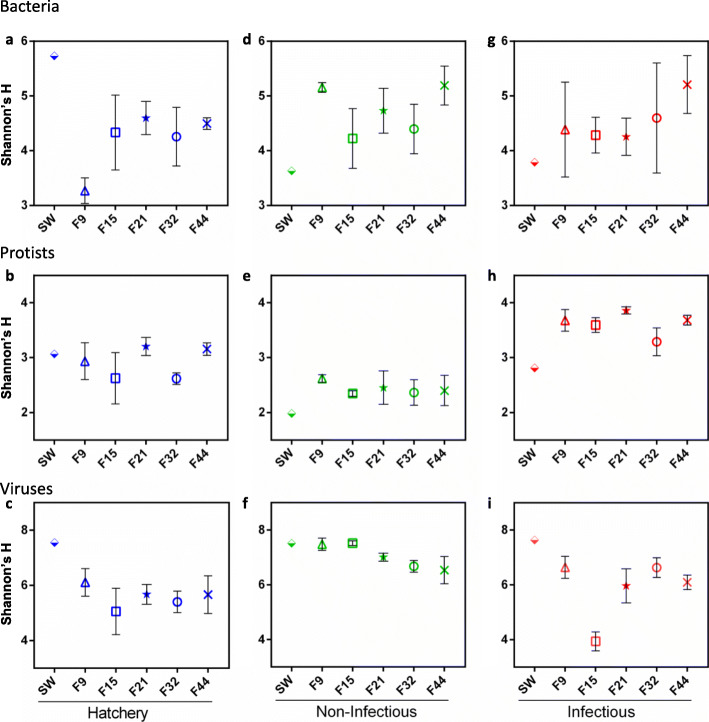
Alpha diversity expressed as Shannon’s H index for bacteria, protists and viruses in the hemolymph and sweater in controlled and natural conditions. **a-c)** Hatchery, **d-f)** natural non-infectious and **g-i)** infectious environment. Bacterial and protistan diversity estimates are based on OTUs, viral on the recovered contigs. The Shannon’s H indices of bacterial **(a, d, g)**, protist **(b, e, h)** and viral **(c, f, i)** communities are presented on the same row. Error bars represent standard error of the mean

**Fig. 4:**
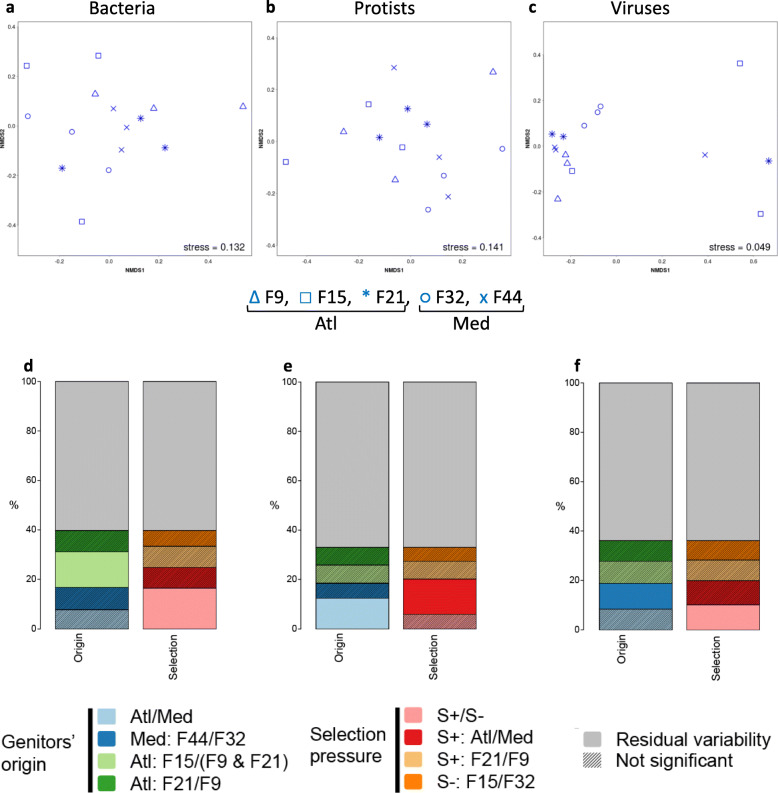
Effect of oyster genotype on hemolymph microbiota. NMDS plots represent beta diversity based on Bray-Curtis dissimilarities for the bacterial (**a**), protistan (**b**) and viral (**c**) communities of oysters born and kept in the hatchery under controlled conditions. Bar plots are graphical representations of Permanova results showing the variation explained by oyster genetic background (genitors’ origin and selection pressure experienced by genitors) for the bacterial (**d**), protistan (**e**) and viral (**f**) communities. Both bars represent the same analysis (model: ~ Family), but with two different sets of orthogonal contrasts (see Additional file 6 Table S5). The terms which are not significant are shown as shaded rectangles (for completeness), but should not be interpreted. Table on which the bar plots are based can be found in Additional File 10: Table S9. Bacterial and protistan diversity estimates are based on OTUs, viral on contigs

To determine if oyster genetic background can influence hemolymph microbiota composition, we compared oysters with different genetic (from the five biparental families) that were maintained in the hatchery from fertilization until the age of one year. The response of hemolymph microbiota to environmental changes is mostly restricted to fine taxonomic scales [44] and we therefore examined the diversity and composition of the hemolymph microbiome at the OTU level (or contig level for viruses). We were particularly interested if the oyster families of the same origin (Atlantic or Mediterranean) and phenotype (resistant S+ and susceptible S-) are more similar to each other despite different genotypes. We therefore defined two sets of a priori contrasts, in order to explore the observed differences from both the aspect of origin and phenotype (Additional file 6 Table S5). Whereas we found no difference in viral alpha diversity between the families, we observed some differences in Shannon’s H indexes for bacterial (F_4,10_ = 6.00, *p* = 0.010) and protist (F_4,10_ = 4.01, *p* = 0.034) communities (Fig. 3a-c; Additional file 9: Table S8). Specifically, differences in bacterial alpha diversity were restricted to resistant families, with the Atlantic resistant families (F9 and F21) having on average lower Shannon’s H than the Mediterranean family (F44) (0.56, 95%CI: (0.137, 0.985)). However, this difference was driven by very low diversity of the F9 family, which was also significantly lower than that of F21, the other Atlantic resistant family (0.66, 95%CI: (0.290, 1.036)). Conversely, the differences in protist Shannon’s H were restricted to Mediterranean families, where the resistant family (F44) had higher diversity than the susceptible one (F32) (0.27, 95%CI: (0.117, 0.419)). Regarding beta-diversity, Permanova analyses revealed that 16% of bacterial and 10% of viral community variability was explained by selection pressure experienced by genitors (Fig. 4a, d and c, f, Additional file 10: Table S9). On the other hand, we found no significant general effect of selection pressure experienced by genitors on the protist communities (S+/S-), while 12% of variability was explained by genitors’ origin (Atl/Med) (Fig. 4b, e, Additional file 10: Table S9). Our findings support the previously reported association between the host genotype and oyster gill and hemolymph bacterial communities [45, 46], which we now extend to protist and viral microbiota.

### Dynamics of microbial consortia during transplantation into a natural environment is mainly shaped by environmental factors

To investigate how the host and environmental factors influence oyster hemolymph microbiota, we compared the microbiota of oyster biparental families after transplantation from the hatchery into the natural environment during non-infectious and infectious periods. Transplantation into natural environments differentially affected the alpha diversity of the three microbial components. According to Shannon’s H index, transplantation did not affect the alpha diversity of bacterial microbiota at all (Fig. 3a, d and g, Additional file 11: Table S10). Although the Shannon’s H of F9 family seems to dramatically increase after the transplantation in the non-infectious environment, neither the interaction terms (environment x genitors’ origin or environment x selection-pressure) nor family random effects were significant, likely due to overall high within-group variability (Additional file 11: Table S10). Average diversity of viral communities increased after transplantation into the non-infectious environment (1.49, 95%CI: (0.977, 2.006)), whereas it remained the same after transplantation into the infectious environment (Fig. 3c, f and i, Additional file 11: Table S10). However, we found significant environment x origin (− 0.59, 95%CI: (− 1.167, − 0.016)) and environment x origin x selection-pressure (0.58, 95%CI: (0.007, 1.158)) interactions for viral microbiota, probably reflecting a large diversity drop in the F15 family during the infectious period. Finally, the average alpha diversity of protist microbiota was lower during the non-infectious period (− 0.45, 95%CI: (− 0.704, − 0.204)) and higher during the infectious period (0.72, 95%CI: (0.467, 0.966)) than in the hatchery (Fig. 3b, e and h, Additional file 11: Table S10). Although we previously observed lower diversity of hemolymph bacterial microbiota coupled with proliferation of opportunistic bacterial pathogens of the genus *Arcobacter* following a *Vibrio sp*. infection [44], we see no link between OsHV-1 μVar infection and bacterial diversity, suggesting that the oyster bacterial microbiota responds differently to bacterial and viral infection.

**Fig. 5:**
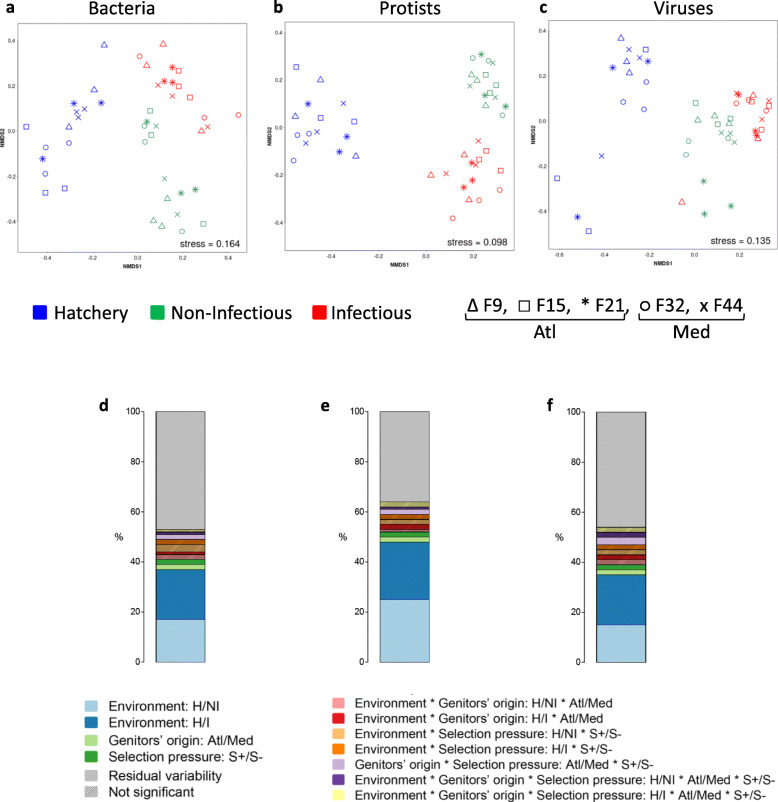
Effect of oysters genetic background and environmental conditions on oyster hemolymph microbiota structure in controlled and natural conditions. NMDS plots of Bray-Curtis dissimilarities for the bacterial (**a**), protistan (**b**) and viral (**c**) communities in the hatchery, and natural non-infectious and infectious environments. Bar plots are graphical representations of Permanova results for the bacterial (**d**), protistan (**e**) and viral (**f**) communities. Permanova model fit here is ~ environment*genitors’ origin *selection pressure (table on which the barplots are based can be found in Additional File 12: Table S11). The terms which are not significant are shown as shaded rectangles (for completeness), but should not be interpreted. Each term is described by the factor or interaction (interactions are marked by *) it represents (i.e. environment, environment * genitors’ origin …), followed by a specific contrast after the colon. Each term is more similar to a linear model coefficient (although it describes the variability explained), and is different from the shared amount of variability explained in redundancy analysis. Bacterial and protistan diversity estimates are based on OTUs, viral on contigs

For all three community types, NMDS plots based on Bray-Curtis dissimilarities revealed grouping according to the environment type (Fig. 5a-c). Permanova confirmed a huge effect of the environment, with 37, 48 and 35% of the variability in bacterial, protist and viral communities, respectively, explained by the environment (Fig. 5d-f; Additional file 12: Table S11), indicating that the composition and dynamics of hemolymph microbial consortia are mainly shaped by environmental factors. This was also supported by the indicator species analysis, as we identified a number of taxa specific to each environment, but found virtually no evidence of origin or selection-pressure signature taxa (Additional File 7:Table S6). Despite the dominant effect of the environment, genitor origin and selection pressure experienced by genitors each explained a low (2–3%) but significant amount of variability in the structure of all three microbial components, suggesting that oyster-related factors do play a role in the microbiota assembly (Fig. 5d-f; Additional file 12: Table S11).

Our results corroborate previous findings showing that the hemolymph bacterial microbiota is mainly influenced by environmental factors [45], and extend these to protists and viruses.

### Trans-kingdom co-occurrence analysis shows disruption of microbial network after transplantation

Co-occurrence network properties can be used to study community stability across different conditions [74, 79, 80]. To assess the influence of the environment on network properties, we constructed separate microbial networks for hatchery, non-infectious and infectious periods. Only bacteria and protists were considered in the network, due to incomplete assembly of viral genomes that can lead to generation of “artefactual networks” (as several contigs may correspond to the same virus). Network comparisons are commonly achieved by calculating the average of four network indices for each node (i.e., taxon): (i) connectivity or degree distribution, representing the number of edges of a node toward other nodes; (ii) path length (the shortest path between two nodes); (iii) clustering coefficient, describing how well a node is connected to its neighbors; and (iv) modularity, a measure of the structuration of the network into modules [81].

**Fig. 6:**
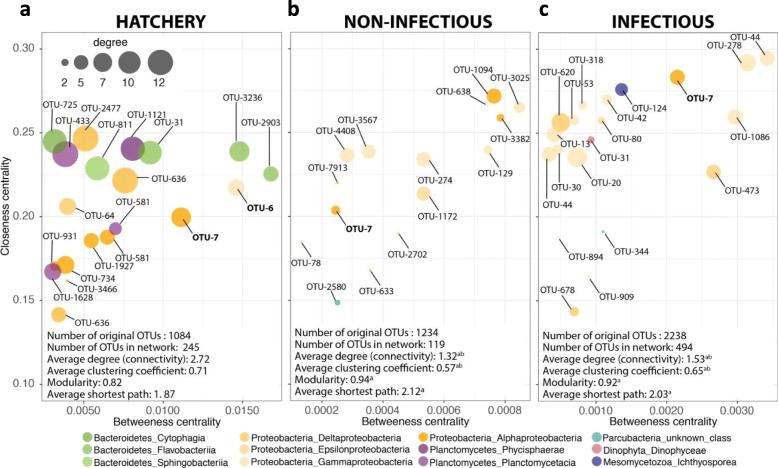
Relationship between closeness and betweenness centrality values for microbial co-occurrence networks in controlled and natural conditions. The plots show closeness vs. betweeness centrality values for nodes in the hatchery (**a**), non-infectious (**b**) and infectious (**c**) networks based on bacterial and protist OTUs. Only the 20 nodes with the highest degree value are represented for each network. Color indicates phylum or class-level taxonomic assignment of the nodes. General network properties are specified in the lower left corner of each plot. A superscript ‘a’ indicates a significant difference (*p* < 0.01) between the hatchery and infectious or non-infectious general network properties. A superscript ‘b’ indicates a significant difference (*p* < 0.01) between infectious and non-infectious general network properties

Overall, we detected significant differences in terms of average connectivity, average shortest path and average clustering coefficient between the environments (Fig. 6a-c, note that x-axes are not on the same scale). Particularly, these results point to a disruption of the microbial network after transplantation into a natural environment during both non-infectious and infectious periods (Fig. 6b and c). Indeed, the higher values for average shortest path in non-infectious and infectious networks may imply lower efficiency and speed in the transmission of information, energy or material in the system, potentially altering the response of the microbial community to environmental perturbations [81, 82]. In addition, the lower connectivity and lower average clustering coefficient indicate a potential perturbation of the network and the loss of important community members [83]. A lower clustering coefficient is indeed an indication of the loss of highly connected nodes (i.e., hubs), which have been related to the concept of keystone species [84, 85].

Calculation of network indices for individual nodes, particularly those related to the concept of keystone species [86], demonstrated this loss. Specifically, nine OTUs from the Bacteroidetes and Planctomycetes phyla scored among the 20 highest values of betweenness centrality, degree and closeness centrality in the hatchery environment, but disappeared in the non-infectious and infectious networks. The loss of such keystone species, the ‘backbone’ of the community [85], could lead to the fragility of the affected network, ultimately leading to its fragmentation and to secondary extinctions of species relying on the hubs [87]. OTUs playing a pivotal role in the structuration of the three networks were mainly dominated by members of the Proteobacteria phylum. OTU-6 (*Gamaproteobacteria, Alteromonadales, Colwelliaceae, Colwellia*) and OTU-7 (*Alphaproteobacteria, Rhodobacterales, Rhodobacteraceae, Planktomarina*) were among the keystone species in the hatchery network, but only OTU-7 was identified in all networks (Fig. 6a-c). Bacteria of the *Roseobacter* clade are known for their probiotic properties and protective effects against *Vibrio* infections in marine vertebrates [88] and invertebrates [89].

### Hemolymph microbiota dynamics in the context of Pacific oyster mortality syndrome

**Fig. 7:**
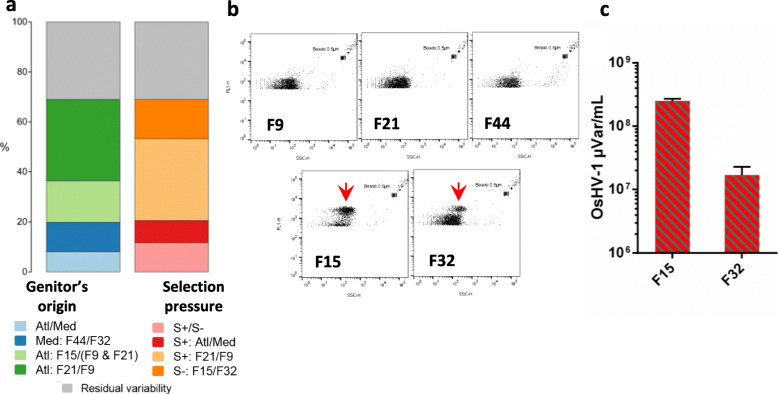
Viral beta diversity and VLP load quantification in oyster hemolymphs during the infectious period. **a** Bar plots are graphical representations of Permanova results showing the amount of variation explained by families grouped by genitors’ origin or selection pressure (for additional explanations see the legend to Fig. 4; table on which the barplots are based can be found in Additional File 13: Table S12). **b** Representative flow cytometry dot plots obtained for resistant (F9, F21 and F44) and susceptible (F15 and F32) families. For families F15 and F32, during the infectious period, we observed an additional VLP population characterized by a higher DNA content (red arrows). **c)** OsHV-1 μVar loads in the oyster hemolymph of families F15 and F32 (mean ∓ SEM)

To evaluate the role of hemolymph microbiota in health and disease, we further investigated the effect of oyster genetic background on β-diversity during the infectious period. Whereas we did not observe any differences in the structure of bacterial communities between the families, we found that selection pressure experienced by genitors explained 10% (*p* < 0.05) of the variability in protist microbiota (Additional file 13: Table S12). Closer inspection revealed that this effect was mainly due to the difference between two Mediterranean families (F32 and F44, Additional file 13: Table S12, compare S+/S- selection pressure contrast and Med: F44/F32 genitors’ origin contrast). For viruses, oyster genetic background explained 69% of the variation in the community structure (Fig. 7a; Additional file 13: Table S12). Interestingly, the differences between Atlantic resistant families accounted for the major part of the variability explained (F9/F21, 33%), which could be explained by the fact that F9 was produced from wild genitors collected in farming area whereas F21 was obtained by a breeding program using mass selection [47, 90]. Still, a considerable portion of variation was explained by the differences between the resistant and susceptible families (S+/S-: 12%; F32/F44: 12%; F15/F9&F21: 16%), as well as between the susceptible families (F32/F15: 16%). Indicator species analysis showed that susceptible families in the infectious environment were characterized by high abundance of OsHV-1 μVar (Fig. 2i; Additional File 7:Table S6) demonstrating the key role of host genetic background in resistance to OsHV-1 μVar as observed in a previous genome-wide association study [91].

To accurately quantify OsHV-1 μVar load within the oyster hemolymph, we used a flow cytometry technique to quantify virus-like particles (VLPs). Flow cytometry dot plots show an additional VPL population in susceptible families (red arrows in Fig. 7b), representing 62.4% (± 1.95 SEM) and 20.3% (± 2.56 SEM) of the overall VLP population in families F15 and F32, respectively. This VLP population was sorted from the hemolymph of the two susceptible families and sequenced; in both cases it corresponded to OsHV-1 μVar (Fig. 7c); corroborating the metagenomic analysis results and thus the role of the genetic background in the control of the OsHV-1 μVar dynamics within oyster hemolymph. In addition, high OsHV-1 μVar concentrations, reaching up to 2.8 × 10^8^ viruses per mL hemolymph, were found only in susceptible families, supporting the previous findings that viral replication occurred in hemocytes [92, 93]. Moreover, we recently showed that the hemocyte infection by OsHV-1 μVar followed by intense replication was an initial and necessary step for disease development in susceptible families, while resistant families successfully controlled viral replication [35]. We hypothesize that the intense viral replication within hemocytes leads to the liberation of OsHV-1 μVar into the circulatory system and its spread to all oyster tissues.

Interestingly, we found no association between oyster susceptibility and the occurrence of particular bacterial taxa. However, oyster colonization by opportunistic bacteria was shown, on the base of whole animal analyses, to be a necessary second step of the infectious process leading to the death of oysters [35], supporting previous findings that identified bacteria as important etiological agent of the disease [48]. The bacteria most frequently associated with oyster mortality belong to the genera *Vibrio, Arcobacter, Marinobacterium, Fusibacter, Psychromonas* and *Psychrobium* [32, 35, 44, 94]. Although the bacteria belonging to these genera characterized oyster hemolymph microbiota during the infectious period, they were not specifically linked to susceptible animals that experienced mortality (Additional File 7: Table S6). However, previous findings were based on solid tissues, whose composition and dynamics differ from those of the hemolymph microbiota [45], which may explain the difference in response to infection. Alternatively, it is possible that these taxa proliferate only during a particular period of disease that we did not cover with our sampling.

## Conclusion

Deciphering the complex interplay between the host, its microbiota and the environment is a vital part of understanding dynamics and outcome of polymicrobial infections, such as the Pacific Oyster Mortality Syndrome. Our study shows that oyster hemolymph is a complex ecosystem strongly influenced by environmental conditions and hosting diverse bacteria, protists and viruses. However, each of these microbiota components is weakly but significantly shaped by oyster genetics, indicating that they indeed interact with the oyster host and that holobiont framework is a helpful concept describing the oyster-microbes community [93]. More studies would be needed to determine if certain microbiota components could be part of the holobiont, potentially affecting fitness of their host, or if they only constitute inconstant environmental component [95].

Interestingly, despite the shared environment, each microbiota components seems to respond differently and independently of the others to environmental conditions. Contrary to previous findings [35, 44, 48, 94], we observed no proliferation of opportunistic bacterial pathogens following the invasion by OsHV-1 μVar, which highlights the need to more closely investigate the mechanisms and dynamics of transkingdom interactions within the hemolymph ecosystem in order to explain this discrepancy.

Finally, the bacterial microbiome displayed stable high-level taxonomic composition and bacteria were the only organisms identified as important in the microbial networks, suggesting a higher stability and more fine-tuned interactions with the oyster host compared to protists. Therefore, it seems that at least some bacteria may be really adapted to the hemolymph conditions, whereas viral and protist communities are primarily transient. However, most of the viral diversity we observe here is dark matter [96] and it is possible that better characterization of oyster-associated viruses may reveal a different picture.

## Supplementary information

**Additional file 1: Table S1.** Overview of specificity of primers used to amplify V1-V2 region of 18S rRNA genes.

**Additional file 2: Table S2.** Bacteria OUT table with sequence tag count per sample and taxonomic affiliations.

**Additional file 3: Table S3.** Protist OUT table with sequence tag count per sample and taxonomic affiliations.

**Additional file 4: Table S 4.** Viral contig table with sequence tag count per sample and taxonomic affiliations.

**Additional file 5: Figure S1.** Sample rarefaction curves of alpha diversity indices for bacterial (**a**) and protists (**b**) datasets. (PPTX 995 kb)

**Additional file 6: Table S5.** Orthogonal contrasts used to examine the differences between the oyster families.

**Additional file 7: Table S6.** Indicator taxa for the oyster environment, family origin and phenotype.

**Additional file 8: Table S7.** Generalized least squares by maximum likelihood linear models testing for differences in alpha diversity between the seawater and hemolymph microbiota within each environment.

**Additional file 9: Table S8.** Generalized least squares by maximum likelihood linear models testing for differences in alpha diversity between the families in the hatchery.

**Additional file 10: Table S9.** Permanova based on Bray-Curtis dissimilarities testing for differences in beta diversity between the families in the hatchery.

**Additional file 11: Table S10.** Linear mixed-effects models testing for effects of the environment, genitors’ origin, selection pressure and their interactions on alpha diversity.

**Additional file 12: Table S11.** Permanova based on Bray-Curtis dissimilarities testing for effects of the environment, genitors’ origin and selection pressure and their interactions on beta diversity.

**Additional file 13: Table 12.** Permanova based on Bray-Curtis dissimilarities testing for differences in beta diversity between the families in the infectious environment.

## Data Availability

The datasets generated during the current study are available in the Sequence Read Archive repository under BioProject ID PRJNA381401 and SRP105754.

## Abbreviations

BIOM: Biological Observation Matrix
FROGS: Find Rapidly OUT with Galaxy Solution
OsHV-1 μVar: *Ostreid herpesvirus* variants
OTU: Operational Taxonomic Unit
POMS: Pacific Oyster Mortality Syndrome
NMDS: Nonmetric Multidimensional Scaling
QIIME: Quantitative Insights into Microbial Ecology
SEM: Standard Error of the Mean
VLP: Virus Like Particle
WGCNA: Weighted Gene Co-expression Network Analysis
