# Supplementary material for: Oyster hemolymph is a complex and dynamic ecosystem hosting bacteria, protists and viruses
Source: Anim Microbiome. 2020 Apr 28;2:12. doi: 10.1186/s42523-020-00032-w (PMC7807429; doi:10.1186/s42523-020-00032-w)
Supplement: Supplementary file 5 — Additional file 5: Figure S1. Sample rarefaction curves of alpha diversity indices for bacterial (a) and protists (b) datasets. (PPTX 995 kb) [file 42523_2020_32_MOESM5_ESM.pptx]

## Slide 1
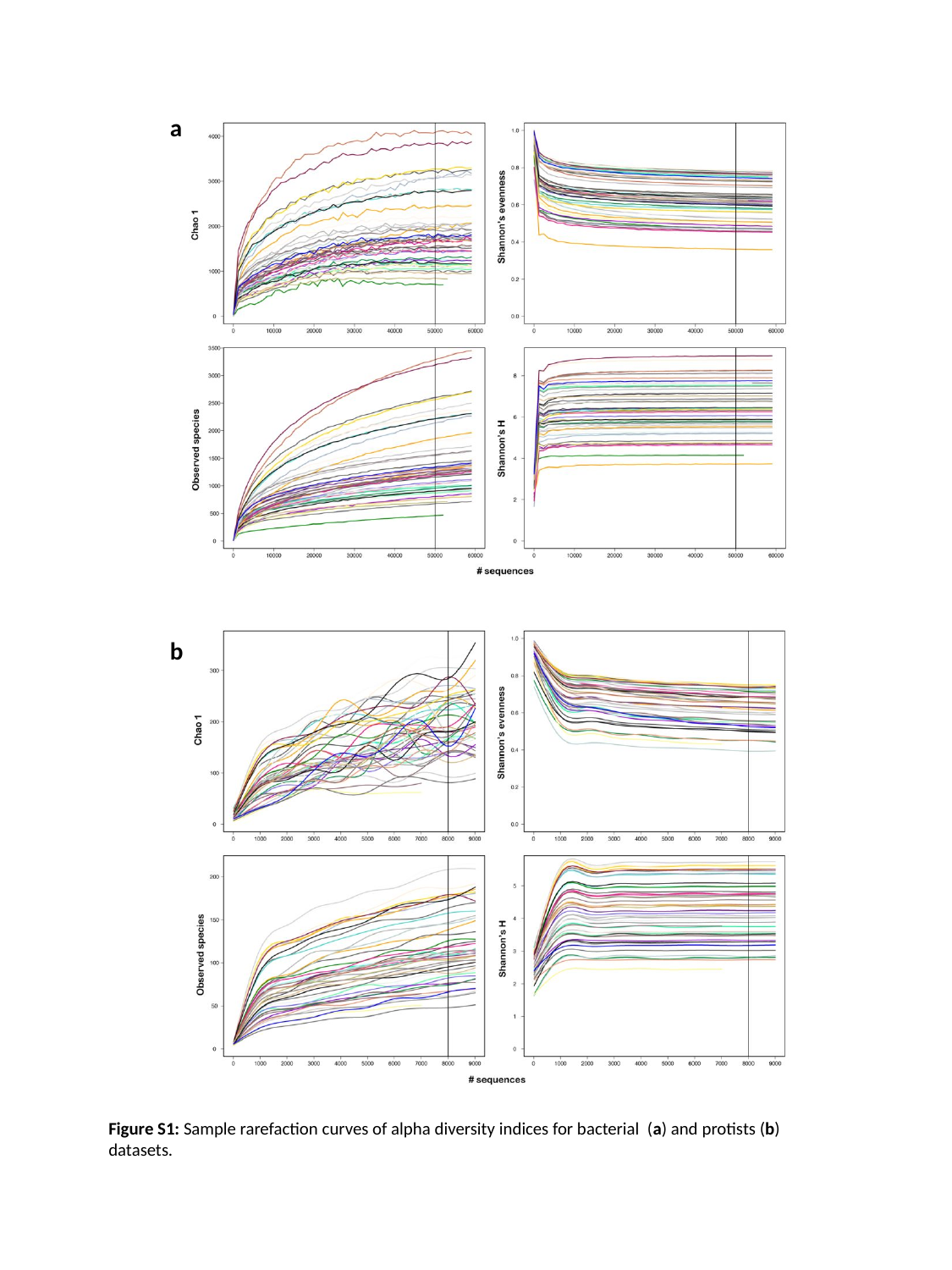

a
b
Figure S1: Sample rarefaction curves of alpha diversity indices for bacterial (a) and protists (b) datasets.
